# Sensitivity of organized convective storms to model grid spacing in current and future climates

**DOI:** 10.1098/rsta.2019.0546

**Published:** 2021-04-19

**Authors:** A. F. Prein, R. M. Rasmussen, D. Wang, S. E. Giangrande

**Affiliations:** ^1^ National Center for Atmospheric Research, 3090 Center Green Drive, Boulder, CO 80301, USA; ^2^ Environmental and Climate Sciences Department, Brookhaven National Laboratory, 98 Rochester St, Upton, NY 11973, USA

**Keywords:** organized convection, mesoscale convective systems, model grid spacing, climate change, rainfall extremes, cold pools

## Abstract

Mesoscale convective systems (MCSs) are complexes of thunderstorms that become organized and cover hundreds of kilometres over several hours. MCSs are prolific rain producers in the tropics and mid-latitudes and are the major cause of warm-season flooding. Traditionally, climate models have difficulties in simulating MCSs partly due to the misrepresentation of complex process interactions that operate across a large range of scales. Significant improvements in simulating MCSs have been found in kilometre-scale models that explicitly simulate deep convection. However, these models operate in the grey zone of turbulent motion and have known deficiencies in simulating small-scale processes (e.g. entrainment, vertical mass transport). Here, we perform mid-latitude idealized ensemble MCS simulations under current and future climate conditions in three atmospheric regimes: hydrostatic (12 km horizontal grid spacing; Δ*x*), non-hydrostatic (Δ*x* = 4, 2 and 1 km) and large eddy scale (Δ*x* = 500 m and 250 m). Our results show a dramatic improvement in simulating MCS precipitation, movement, cold pools, and cloud properties when transitioning from 12 km to 4 km Δ*x*. Decreasing Δ*x* beyond 4 km results in modest improvements except for up- and downdraft sizes, average vertical mass fluxes, and cloud top height and temperature, which continue to change. Most important for climate modelling is that Δ*x* = 4 km simulations reliably capture most MCS climate change signals compared to those of the Δ*x* = 250 m runs. Significantly different climate change signals are found in Δ*x* = 12 km runs that overestimate extreme precipitation changes by up to 100%.

This article is part of a discussion meeting issue ‘Intensification of short-duration rainfall extremes and implications for flash flood risks’.

## 1. Introduction

Mesoscale convective systems (MCSs) play an important role in the earth’s energy balance [1,2] and are essential for the water cycle in the tropics [3] and mid-latitude regions [4,5]. These systems are prolific rain producers and are the main cause of warm-season flooding [6,7]. Observations of MCSs over the continental USA indicate that extreme precipitation rates associated with MCSs have significantly increased since the 1980s [4], and MCSs are projected to further intensify under future climate change scenarios [8]. A major bottleneck for predicting possible climate change effects on future extremes is that convective storms and storm intensity (precipitation, updraft strength) are poorly represented by state-of-the-art models [9]. These challenges are exacerbated for MCS that represent some of the largest and most impactful of convective storms. This is because MCSs entail processes that operate and interact across a wide range of scales, which makes them hard to constrain with limited observations [1,10]. Improving MCS modelling capabilities is essential to advance the credibility of weather predictions and climate projections, especially for socioeconomic impactful extreme events (e.g. floods, droughts).

The frontier of global and regional atmospheric modelling has reached convection-permitting scales (horizontal grid spacings Δ*x* ≤ 4 km) [11]. Convection-permitting models (CPMs) can explicitly represent deep convection, which revolutionizes our ability to simulate and predict the weather and climate system [11,12]. CPMs substantially improve the simulation of MCSs including their propagation, evolution, size and associated extreme precipitation [13]. This paper provides a multi-scale analysis of why this is true. An ongoing challenge of kilometre-scale modelling is that these models operate in the grey zone of turbulent motion, wherein convection is not fully resolved [14]. This causes challenges in realistically simulating cloud entrainment processes and draft characteristics [15].

Simulations in the turbulent grey zone truncate the turbulent energy spectrum, leading to misrepresentations of convection dynamics, which can result in a factor of two overestimation of convective updraft intensity in CPMs [16,17]. Moreover, the energy spectrum of deep convective clouds is continuous across kilometre to metre scales, without an apparent energetic gap indicating a scale separation [18]. Thus, choosing an appropriate grid spacing to realistically simulate deep convective clouds is difficult, since spatial structures of turbulent motion do not converge until metre scales [15]. Although turbulent motions are not fully resolved at the kilometre scale [19], several studies have demonstrated that convergence of convective storm bulk properties (e.g. precipitation accumulations over a mesoscale region) can be achieved with kilometre-scale models [20–23]. A better understanding of the impacts of simulating in the grey zone is paramount since the climate community is rapidly transitioning to kilometre-scale grid spacings whereas large eddy simulations on climate time scales are far out of reach [11].

In this study, we address two main research questions:

(i) How are processes that interact within an MCS simulated across Δ*x* spanning two orders of magnitude from hydrostatic scales (Δ*x* = 12 km) to large eddy scales (Δ*x* = 250 m)?
(ii) Which Δ*x* is needed to reliably simulate MCS process changes under global warming?

The first question aims to identify systematic differences between large eddy simulations (Δ*x* = 250 m) and grid spacings that are currently tested for regional and global climate modelling (Δ*x* = 4 km and Δ*x* = 12 km). The main objective is to investigate the convergence of bulk MCS properties. Identifying grid spacings that can reliably capture salient MCS properties such as total precipitation, vertical mass transport and the cloud shield properties is important to capture the global energy budged and hydrological cycle at efficient computational costs. The second question assesses the robustness of climate change signals comparing results from large eddy simulations with simulations with grid spacings that are currently feasible for climate modelling.

The novel contribution of this study is the use of 10 member ensemble simulations in current and future climate conditions under business as usual warming. Using an ensemble-based approach allows investigating how systematic Δ*x*-dependent differences are to changes in MCS inflow environments. Furthermore, we use a larger domain than previous studies, enabling realistic simulation of three-dimensional MCSs, rather than MCS sections in a channel configuration [15,24].

## 2. Data and methods

The programmes that were used for data processing and visualization in this paper are available on GitHub [25].

### (a) Initial sounding for idealized simulations

The initial conditions for running idealized simulations are based on inflow soundings of air that gets advected into heavy precipitating MCSs. The soundings are derived from two 13 year long climate simulation covering most of North America under current and future climate conditions [26]. Current and future climate simulations were performed using the Weather Research and Forecasting (WRF) model [27,28] with a grid spacing of Δ*x* = 4 km. At this resolution, deep convection can be explicitly represented in the model without the need for a deep convection parametrization [11,23]. The current climate condition simulation downscales ERA-Interim reanalysis data [29] within the period from October 2000 to September 2013. The future simulation uses the pseudo global warming approach [26,30,31] by adding monthly climate change perturbations to the 6-hourly boundary conditions of ERA-Interim during the same time period. The perturbations are derived from an ensemble of CMIP5 (fifth phase of the Coupled Model Intercomparison Project) global climate model projections [32]. These models use the high-end representative concentration pathways (RCP8.5) comparing the period 2071–2100 to 1976–2005. More information about these Δ*x* = 4 km climate simulations are found in Liu *et al.* [26].

Inflow environmental conditions are derived from these simulations by using the identification of MCSs from our previous study [13]. All MCSs that are identified in the central US warm season (June, July and August; JJA) are ranked according to their peak hourly rainfall rate. MCS inflow environments are derived from 3 hourly model level output and are defined as the MCS relative upstream region that is perpendicular to the largest equivalent potential temperature gradient [33,34]. Within the inflow environment, we search for grid cells that have large maximum convective available potential energy (CAPE), low convective inhibition (CIN) and large precipitable water in radial bands of 30 km centred on the location of maximum precipitation with a maximum radius of 320 km. We excluded more distant sounding locations to capture inflow air properties that are affecting the actual MCS development. The environmental variables are calculated from mean air parcel condition (e.g. temperature, moisture) within a depth of 500 m centred on the maximum equivalent potential temperature level in the lowest 3 km above the surface. To exclude inflow grid-cells that are contaminated by the MCS or other precipitating clouds, we remove all cells that are closer than 40 km to grid cells with precipitation (precipitation rates >0.01 mm h^−1^). These filtering steps retain several optimal inflow grid cells for which various diagnostics are calculated (electronic supplementary material, figure S1). We manually investigate these diagnostics and select suitable soundings to initialize idealized WRF simulations.

### (b) Model set-up

We use the WRF model v. 3.9.1.1 to perform idealized MCS simulations. The source code of WRF is available from GitHub (https://github.com/wrf-model/WRF). The model set-up is adapted from the WRF tutorial idealized case three-dimensional supercell thunderstorm, which is called em_quarter_ss [35]. A single sounding provides the initial and boundary conditions that are kept constant over time. Domain size sensitivity tests showed that MCS features such as accumulated precipitation start to converge at domain sizes of 600 × 600 km or larger (not shown). We use 95 vertical levels with an equal distance of 250 m similar to [15] and a 620 km^2^ horizontal domain. Limited sensitivity to decreases in vertical grid spacing has been shown for an idealized squall line case [15]. We use open boundaries and apply Rayleigh damping to the top 20-levels of the model domain to avoid wave reflections.

In all simulations, we neglect the effects of radiation, surface fluxes, Coriolis acceleration and do not use a planetary boundary layer scheme. Surface drag is included by applying the Eta surface layer scheme [36], which helps to form coherent cold pools that organize convection. The Thompson microphysics scheme is used [37], which was also applied in the climate simulations from which the initial MCS inflow soundings are derived [26]. This scheme has been shown to result in high-quality MCS simulations in the central USA [38]. The Kain–Fritsch deep convection scheme [39] is only used in one of the Δ*x* = 12 km simulations. In those simulations, we test the effect of using the Kain–Fritsch deep convection closure [39] in addition to explicitly simulating deep convection. An overview of the performed simulations is shown in table 1.

**Table 1.** Set-up of model simulations. *Nx* and *Ny* denote the grid cells in the longitude and latitude direction. All simulations use the Thompson microphysics scheme [37], the Eta surface layer scheme [36] and have 95 vertical levels with 250 m equal distance.

	large eddy		kilometre scale			hydrostatic	
Δ*x*	250 m	500 m	1 km	2 km	4 km	12 km	12 km C
*Nx*/*Ny*	2495/2495	1247/1247	623/623	311/311	155/155	51/51	51/51
Δ*t* (s)	1	2	4	4	6	10	10

All MCSs are simulated at six horizontal grid spacings: Δ*x* = 12 km, 4 km, 2 km, 1 km, 500 m and 250 m. At Δ*x* = 250 m, the model starts to resolve entrainment/detrainment [15]. Convection is initiated by using a similar approach to previous work [15,40] by forcing vertical motion within a half elliptic cylinder with a length of 40 km, a radius of 20 km and a depth of 4 km. The flat side of the half-cylinder is located at the surface. We use a maximum vertical acceleration of 2 m s^−2^ along the centre of the cylinder. The acceleration decays with the cosine of the radius. We randomly perturb potential temperature by 0.1 K in a rectangular area with a size of 110 × 80 × 4 km centred on the half-cylinder to facilitate the development of three-dimensional motion. This approach is used in all simulations but the location of the half-cylinder was adjusted depending on the MCS movement in each simulation to trigger convection close to the inflow boundary to maximize the time before the MCS reaches the outflow boundary. We performed sensitivity tests using warm bubbles instead of a convergence area to trigger convection, which frequently did not result in the development of deep convection. This is likely because most mid-latitude MCS are baroclinic/synoptically driven, and not primarily forced by radiational heating.

We test 36 current climate condition soundings and 46 future climate soundings at Δ*x* = 4 km to investigate the development of MCSs. This grid spacing is sufficient to assess if the sounding results in the development of an MCS. A larger number of sounding had to be tested in the future climate because fewer soundings supported the development of organized convection mainly because of a reduction of low-level relative humidity. Based on visual inspection, we select 17 cases in each climate that develop an MCS and rank them according to their peak hourly rainfall rate. Members 4–14 are selected in both periods and simulated using all six horizontal grid spacings. This is done to increase the robustness of the climate change analysis by excluding atypically strong and weak MCSs. Including high-end extreme events in the analysis would increase the impact of chaotic processes on our climate change assessment and would demand a much larger ensemble of MCSs than we can afford with our available computer resources [41]. The 10 current and future soundings that can be used to initialize idealized WRF simulations can be accessed from https://issues.pangaea.de/browse/PDI-23519.

### (c) MCS processes

**Figure 1.**
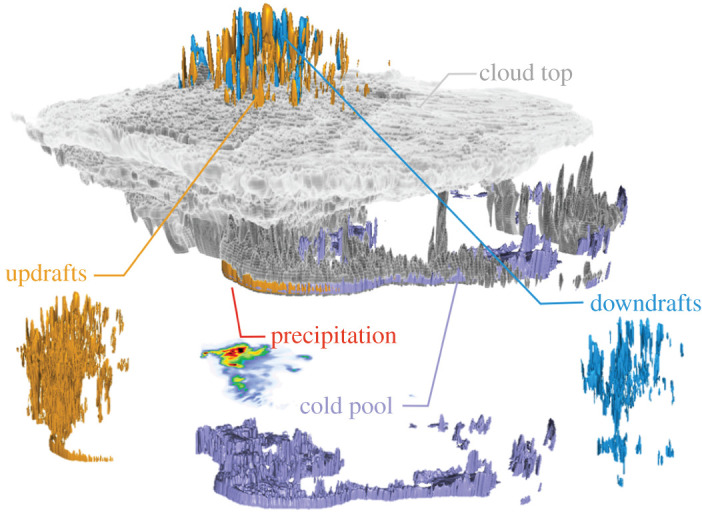
Volume rendering of an example Δ*x* = 250 m MCS cloud field and key MCS components. The vertical extent of the MCS is stretched by a factor of 10. (Online version in colour.)

All analyses are mainly performed on the common grid of the Δ*x* = 12 km simulations unless otherwise noted. Conservative remapping [42] was used to ensure the conservation of mass and energy. Comparing the simulations on the same grid helps to assess processes on similar scales. However, this approach averages out small-scale features in the higher resolution simulations that might be important for specific applications (e.g. hail formation or local scale flood assessments). Therefore, the models are also compared on their native grid concerning the characteristics of convective up- and downdrafts and climate change impacts on precipitation. The salient MCS components that are evaluated are shown in figure 1.

We use an object-based approach for our analyses. This involves thresholding the spatio-temporal data to create a binary field in which objects can be identified. Thereafter, we consider coherent precipitation areas that are connected in space and time as an object—which we refer to as MCS—similar as in our previous work [8,13]. This allows focusing our analysis solely on the MCS by excluding potential secondary convection in the domain. Only objects that do not contact the domain boundaries are considered. Furthermore, for convergence assessments, we only evaluate data 3 hours after model initialization. At this time, MCSs have reached a mature stage (rainfall area, integrated vertical mass flux) and Δ*x*-dependent differences of MCS processes are constant or demonstrate only slow and gradual changes. The only exception are anvil cloud properties, which are evaluated 1 h after simulation start since anvil clouds reach the domain boundary typically within the first 4 h. This improves the robustness of the analysis but results are similar if anvil clouds properties are evaluated between hour 3 to 4. We suggest the simulations to be ‘converged’ when the differences between simulations at smaller Δ*x* are non-systematic and insignificant, which is the case after 3 h. We do not analyse characteristics that are associated with the MCS size (e.g. total precipitation, size of the anvil cloud, cold pool extent) because these characteristics can strongly depend on the initialization of the MCS and vary from real cases that are typically associated with an atmospheric boundary (i.e. synoptic scale forcing).

#### (i) Convective and stratiform precipitation

To differentiate between the MCS and potential secondary precipitating storms that develop remotely from the MCS, we select the largest contiguous precipitation region with precipitation larger than 0.1 mm h^−1^. The MCS convective and trailing/detrained startiform precipitation regions are differentiated by the area with radar 2 km reflectivity more than 40 dBZ. This is in accordance with previous radar-based observations [43,44].

#### (ii) Draft geometry and dynamics

We identify drafts within each MCS as three-dimensional objects of adjacent (horizontally, vertically and diagonal) grid cells with at least 3 m s^−1^ vertical wind speed for updrafts and −3 m s^−1^ for downdrafts [45]. Only drafts with more than 20 dBZ average reflectively, below 16 km, and above an area with precipitation of at least 2.5 mm h^−1^ are analysed to not include clear air vertical motions (e.g. gravity waves) in our analysis. This method is adapted from observational studies using vertical pointing radar for wind profiling [46]. Draft statistics are performed on the native grid to estimate structural convergence of core properties. We randomly selected up to 50 cores per output time step (5 min) to reduce the computational costs of the analysis for sub-kilometre-scale simulations, which can have several hundred cores.

#### (iii) Cold pools

The spatio-temporal evolution of cold pools is captured with an approach that was used in previous studies [47]. Buoyancy near the surface (*b*; m s^−2^) is calculated following [48]:

2.1 $$b=\frac{g\cdot (\Theta_{p}-{\bar{\Theta }}_{p})}{{\bar{\Theta }}_{p}}$$

with *g* being the gravitational acceleration (*g* = 9.81 m s^−2^), *Θ*_*p*_ (K) is the virtual potential temperature, and the overbar indicates a 100 km × 100 km moving average low-pass filter. *Θ*_*p*_ is defined as

2.2 $$\Theta_{p}=\Theta \cdot (1+0.608\cdot Q_{\text{vapour}}-Q_{\text{cloud}}-Q_{\text{rain}}),$$

where *Θ* is the potential temperature in Kelvin, and *Q*_vapour_, *Q*_cloud_ and *Q*_rain_ are the mass mixing ratios of water vapour, cloud condensate and rain water in kg kg^−1^, respectively. In accordance with previous studies [24,49], we calculate the cold pool intensity (*B*; m s^−1^) as

2.3 $$B=\sqrt{-2\int_{0}^{h}b\;\text{d}z}$$

with buoyancy *b* (equation (2.1)) integrated from the surface to *h*, which is the height at which *b* first exceeds −0.005 m s^−2^. Some of the cold pools are directly connected to downdrafts and can, therefore, reach into the mid-troposphere. To reduce computational resources, we limited the maximum height of cold pools to 3.875 km, which has no significant impact on our results.

#### (iv) Anvil cloud properties

We define the anvil cloud as the contiguous three-dimensional area where the sum of the ice mass mixing ratio (*Q*_ice_) and the snow mass mixing ratio (*Q*_snow_) is larger than 0.1 g kg^−1^. The anvil properties investigated are the average cloud top height and the corresponding cloud top temperature. Cloud top characteristics are analysed between hour 3 to hour 5 after model initialization to avoid including statistics when large parts of the anvil are outside the model domain (typically after hour 5).

## 3. Results

**Figure 2.**
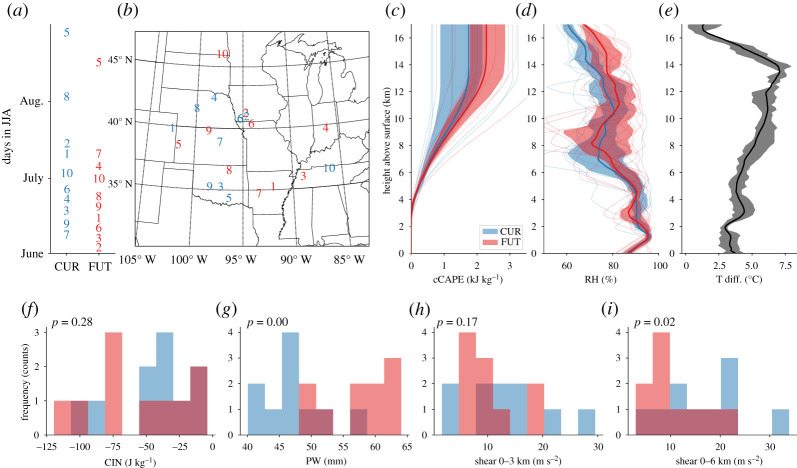
Date (*a*), location (*b*) and characteristics (*c*–*i*) of current (blue) and future climate (red) MCS inflow soundings that are used to initialize idealized simulations. Thin lines show the cumulative convective available potential energy (cCAPE; *c*) and relative humidity (RH; *d*) of individual soundings, thick lines show the ensemble mean and contours show the ensemble interquartile spread. We show the mean air temperature difference between future and current climate soundings (Tdiff.; *e*) as a black bold line and the interquartile range as a grey contour. Convective inhibition (CIN; *f* ), precipitable water (PW; *g*), bulk wind shear between the surface to 3 km (*h*) and 6 km height (*i*) are shown in histograms.The significance of differences between the future and current inflow soundings is indicated by the two-sided *p*-values of a Mann–Whitney rank test (P; *f* –*i*). (Online version in colour.)

In figure 2, we show properties of the 10 inflow sounding that are used to initialize the idealized current and future climate MCS simulations. Most soundings were derived from MCSs in the first half of JJA. There is a slight shift to earlier sampling dates in the future climate (figure 2*a*). The sounding locations are randomly sampled throughout the central USA (figure 2*b*). Note that cumulative CAPE (cCAPE, figure 2*c*) tends to increase in the future soundings at high altitudes. However, approximately below 7 km the soundings have similar cCAPE values. The CIN properties (figure 2*f* ) do not change significantly between current and future climate scenarios, and the same behaviour holds for the relative humidity (RH; figure 2*d*). Constant relative humidity means that the atmospheric precipitable water (PW; figure 2*g*) increases at close to Clausius–Clapeyron rates (approx. 6.5% per degree warming [50]). Air temperature increases roughly twice as fast at high-levels (approx. 7°C at 14 km; figure 2*e*) than near the surface (approx. 3.5°C), resulting in a stabilization of the troposphere and an enhanced moistening of upper levels compared to low levels. Ground to low-level wind shear (figure 2*h*) does not change significantly, while median mid-level shear (figure 2*i*) is on average 5 m s^−1^ smaller in future climate soundings. The modelled soundings that we use here are comparable to observed pre-MCS soundings at the US Department of Energy’s Atmospheric Radiation Measurement Southern Great Plains site in Oklahoma except for higher low-level RH and lower CIN values in the model soundings [46,51].

### (a) Grid spacing dependencies under current climate conditions

In this section, we present results addressing our first research question on how MCS components are simulated when using horizontal grid spacings that range from hydrostatic to large eddy scales. We use an ensemble of 10 idealized MCS simulations to understand the robustness of our results to different MCS environments. All figures in this section feature a representative MCS example to illustrate grid spacing dependent differences followed by ensemble-based analysis.

#### (i) Precipitation characteristics

In this section, we assess key precipitation characteristics from the 10 member current climate MCS ensemble to understand if there are systematic scale-dependent differences. Note that all ensemble evaluations are performed on a common 12 km grid unless otherwise noted.

**Figure 3.**
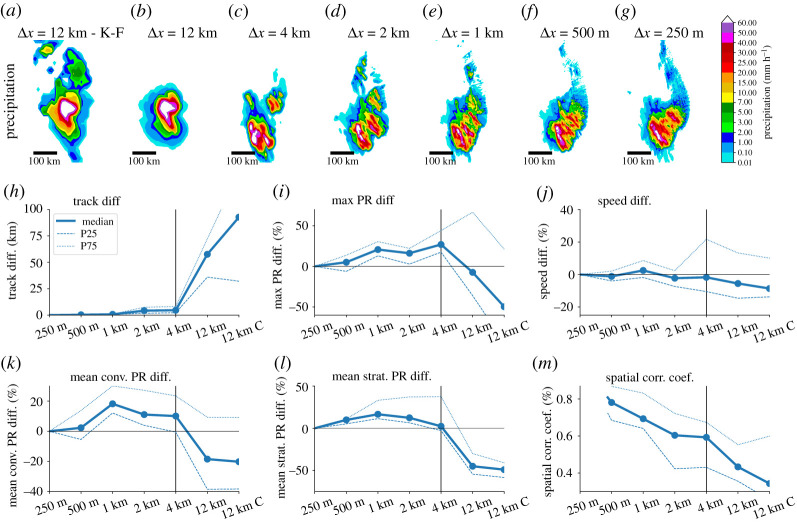
Hourly precipitation accumulation (4.40–5.40 after simulation start) of an example MCS case under current climate conditions showing the sensitivity to the model grid spacing from Δ*x* = 12 km (left) to Δ*x* = 250 m (right) (*a*–*g*). Relative differences in MCS track distance (*h*), peak precipitation (*i*), movement speed (*j*), mean convective (*k*), and mean stratiform precipitation intensity (*l*) between current climate MCSs compared to their Δ*x* = 250 m counterpart. Five minute model output from mature MCSs (3-h after initiation and before they reach the domain boundary) are considered. (*m*) Spatial correlation coefficient of the MCS total precipitation (accumulated over hour 3 to 7) between coarser simulations and their Δ*x* = 250 m counterpart. Correlation coefficients are maximized by shifting the precipitation patterns to reduce penalties due to spatial displacements. (*h*–*m*) Thick lines show the ensemble median, dashed lines the 25 percentile and dotted lines the 75 percentile. (Online version in colour.)

MCS precipitation features can change significantly contingent on Δ*x*, as shown on the example of hourly MCS precipitation accumulation in figure 3*a*–*g*. There is a regime shift in the spatial structure and location of precipitation when transitioning from hydrostatic (Δ*x* = 12 km) to non-hydrostatic (Δ*x* ≤ 4 km) grid spacing. Further increasing Δ*x* results in the addition of small-scale variability and a northward extend of stratiform rainfall but no fundamental changes in mesoscale structures in the intense precipitation region.

Figure 3*h*–*m* shows the ensemble mean and variability of precipitation properties from coarser Δ*x* simulations compared to their Δ*x* = 250 m counterpart. The location of the MCS track is remarkably similar (within less than 10 km displacement) for simulations with grid spacings up to 4 km. However, significant track discrepancies occur in the Δ*x* = 12 km simulations (figure 3*h*) with smaller differences in those without deep convection scheme.

Maximum hourly precipitation accumulations suggest a clear regime shift when transferring non-hydrostatic to hydrostatic simulations with the latter simulations typically showing lower precipitation intensities and much less accuracy (large ensemble spread; figure 3*i*). Maximum precipitation is systematically approximately 20% larger using Δ*x* = 4 km to Δ*x* = 1 km, which is consistent with previous results [52,53]. A similar behaviour can be seen for mean convective (figure 3*k*) and stratiform (figure 3*l*) precipitation with the latter showing substantial low biases of approximately 50% in the Δ*x* = 12 km simulations due to the lack of a stratiform shield. MCSs movement speed does not show a strong grid spacing dependence (figure 3*j*).

Next we analyse how similar the spatial pattern of the total MCS accumulated precipitation (hereafter precipitation footprint) are compared to those of the Δ*x* = 250 m simulation. To avoid penalties from displacement errors, we shift the precipitation footprint from the coarser resolution simulations relative to the footprint of the Δ*x* = 250 m run until the spatial correlation coefficient is maximized. We see a decrease of correlation coefficients from 0.8 for Δ*x* = 500 m to 0.6 at Δ*x* = 2 km. Δ*x* = 2 km and Δ*x* = 4 km simulations have similar correlation coefficients while Δ*x* = 12 km show correlation coefficients around 0.4.

In summary, there is a clear regime shift in simulating MCS precipitation characteristics when transitioning from non-hydrostatic to hydrostatic scales. The latter have significantly lower skill in capturing the precipitation location, intensity and spatial patterns simulated by the Δ*x* = 250 m runs. Additionally, there are clear benefits of not using the Kain–Fritsch deep convection parametrization at Δ*x* = 12 km in the simulated precipitation characteristics. From our simulations, it is unclear how intermediate grid spacing simulations using Δ*x* = 6 km or Δ*x* = 8 km would perform, which should be the focus of future studies.

#### (ii) Vertical mass flux and draft geometry and dynamics

**Figure 4.**
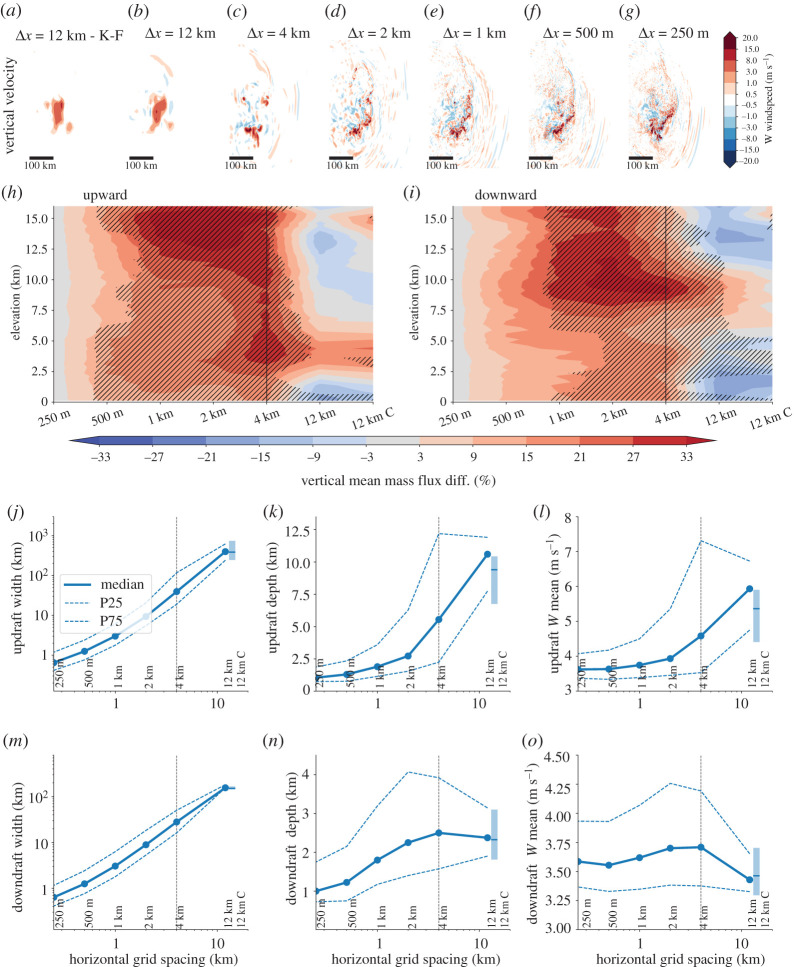
MCS up- and downdraft characteristics dependence on horizontal grid spacing. (*a*–*g*) Vertical velocity at 5 km height above surface for the same MCS case as in figure 3*a*–*g* 4 h and 40 min after simulation start. Accumulated current climate MCS wide upward (*h*) and downward (*i*) mass flux differences relative to the Δ*x* = 250 m simulation based on 5 min output between hour 3 to hour 7 after simulation start. Up- (*j* and *l*) and downdraft (*m* and *o*) width (*j* and *m*), depth (*k* and *n*), and mean velocity dependence on model horizontal grid spacing in current climate MCSs. Thick lines show the ensemble median and dashed/dotted lines the 25/75 percentile. Box whisker plots show results from the Δ*x* = 12 km simulations with deep convection parametrization. This analysis is performed on the native model grid. (Online version in colour.)

The impact of horizontal model grid spacing on vertical wind speed at mid levels is shown for one example MCS in figure 4*a*–*g*. The Δ*x* = 250 m simulation shows high spatial variability with small but intense vertical up- and downdrafts along the leading edge of the MCS. Additionally, there are gravity waves propagating ahead of the MCS. These characteristics are qualitatively captured even in the Δ*x* = 4 km run but the up- and downdraft sizes are larger and less variable. In the Δ*x* = 12 km runs, the MCS collapses into one dominating updraft and gravity waves are largely absent especially in the simulation with deep convection parametrization.

The ensemble average mass flux in the MCSs is overestimated in kilometre-scale models compared to the Δ*x* = 250 m simulations (figure 4*h*). Δ*x* = 1 km and Δ*x* = 2 km simulations have an overestimating of more than 33% close to the cloud top while the Δ*x* = 4 km runs overestimates mass flux by about 20% above 3 km height. The Δ*x* = 12 km simulations have more similar mass flux statistics compared to the Δ*x* = 250 m than the kilometre-scale simulations, which is likely due to error cancellation effects (e.g. much larger updrafts with lower vertical wind speeds). Average downward mass flux statistics show similar Δ*x* dependencies compared to upward mass fluxes (figure 4*i*). Note that the maximum overestimation in the downward mass flux occurs at around 10 km height, which is connected to the maximum overestimation in the upward statistics that occurs above this level. The enhanced mass flux in kilometre-scale models is likely related to a underestimation of entrainment and detrainment due to an under-representation of small-scale turbulence [15,19].

The structural convergence of core properties is investigated on the native model grid. Up- and downdraft characteristics change substantially with Δ*x*. At Δ*x* = 12 km, approximately 200/150 km wide (figure 4*j*,*k*) and 10/2 km deep up/downdrafts (figure 4*m*,*n*) are simulated. Updraft width decreases exponentially with Δ*x*, but start to flatten towards Δ*x* = 250 m, indicating potential convergence at a higher resolution. This flattening is less pronounced for downdraft widths, meaning that structural convergence of downdraft width demands smaller Δ*x* than updraft convergence.

Updrafts are plume-like (a rising column of warm air) in the Δ*x* = 12 km simulations with an average depth of 10 km (figure 4*k*), which is consistent with our previous study [46]. Decreasing Δ*x* makes updraft more thermal-like (rising bubbles of warm air) with a mean depth of 1.25 km at Δ*x* = 250 m. Mean updraft depth decreases rapidly between Δ*x* = 12 km and Δ*x* = 2 km and starts to flatten afterwards. By contrast, mean downdraft depth is similar between Δ*x* = 12 km and Δ*x* = 2 km, starts to decrease from Δ*x* = 2 km to Δ*x* = 500 km, and flattens afterwards (figure 4*n*). In previous work, we compare a subset of the here used simulations to radar wind-profiler observations and show that the sub-kilometre-scale simulations significantly improve the representation of draft geometry [46].

Mean updraft speed is 6 m s^−1^ in the Δ*x* = 12 km simulations, which is almost twice as fast as in Δ*x* ≤ 250 m runs (figure 4*l*). The overestimation in the Δ*x* = 12 km simulations of 4.5 m s^−1^ is already substantially improved in Δ*x* = 4 km runs and starts to converge at Δ*x* ≤ 2 km. Average downdraft speed is generally less scale sensitive than updrafts speed (figure 4*o*). Here, Δx = 12 km simulations clearly underestimate the velocity while average downdraft speed does not change significantly in simulations with Δ*x* ≤ 4 km.

#### (iii) Cold pool properties

**Figure 5.**
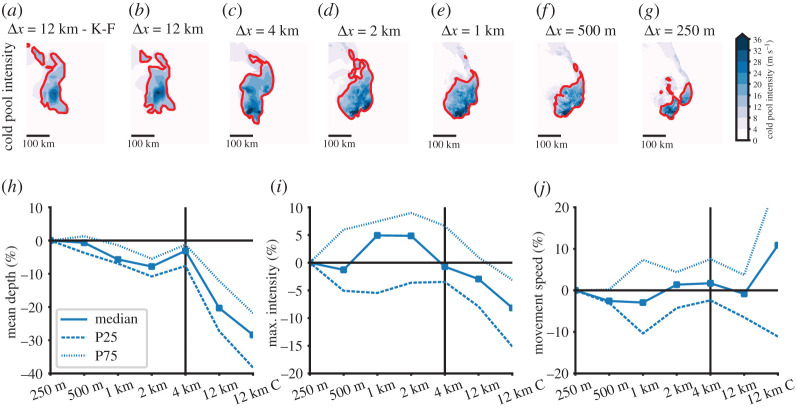
MCS cold pool characteristics dependence on horizontal grid spacing. (*a*–*g*) Cold pool intensity for the same MCS case as in figure 3*a*–*g* 4 h and 40 min after simulation start. The red contour line shows the part of the coldpool that was used for the analysis in (*h*–*j*). Average cold pool depth (*h*), maximum intensity (*i*) and movement speed (*j*) differences relative to the Δx = 250 m simulations. Thick lines in *h*–*j* show ensemble median differences and dashed/dotted contours show the 25/75 percentile base on 5 min model output.(Online version in colour.)

Figure 5*a*–*g* shows the cold pool intensity (see Method section for the definition) of an example MCS at different horizontal grid spacings. Similar to the precipitation analysis, cold pools also show a clear displacement when decreasing the Δ*x* from 4 km to 12 km. This is understandable since these idealized simulations are cold pool driven (e.g. see the development of updrafts along the leading edge of cold pools in figure 1). It is also obvious that the intense part of the cold pool becomes smaller with increasing Δ*x* and that the location of the intense regions is closer to the middle of the cold pool in the Δ*x* = 12 km simulations, whereas it is at the leading edge of the cold pool in the higher resolution runs.

The mean cold pool depth is systematically smaller by up to −10% in kilometre-scale simulations compared to the Δ*x* = 250 m runs (figure 5*h*). Much larger underestimations of −30/−20% occur in the Δ*x* = 12 km simulations with/without deep convection parametrizations. Maximum cold-pool intensities are well captured up to Δ*x* = 4 km and are systematically lower in the Δ*x* = 12 km runs (figure 5*i*). The mean movement speed of cold pools is well captured across all grid spacings but the ensemble spread is significantly larger in the Δ*x* = 12 km with deep convection scheme (figure 5*j*). As expected, the movement of the cold pools is similar to the movement of the precipitation area that was discussed earlier (figure 3*j*).

#### (iv) Anvil clouds and hydrometeor properties

**Figure 6.**
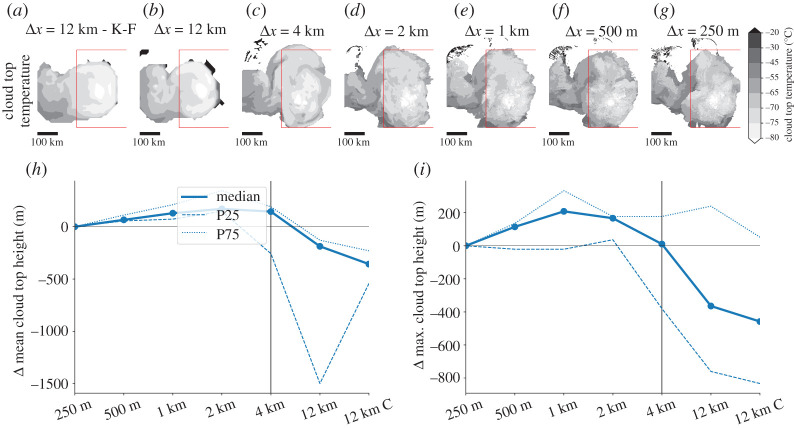
(*a*–*g*) Cloud top temperatures for the same MCS case as in figure 3*a*–*g* 4 h and 40 min after simulation start. The red box shows the area of the MCS that is shown in figure 3–5*a*–*g*. Average (*h*) and maximum (*i*) cloud top height differences relative to the Δ*x* = 250 m simulations. Thick lines in *h,i* show ensemble median differences and dashed/dotted contoursshow the 25/75 percentile base on 5 min model data 1 h after simulation start. (Online version in colour.)

Figure 6*a*–*g* shows cloud top temperatures from an example MCS across grid spacings. Similar to previous analysis, there is a clear change in the spatial structure and average cloud top height when increasing Δ from 4 km to 12 km. This is also obvious in the ensemble mean cloud top height statistics (figure 6*h*). Kilometre-scale simulations slightly overestimate the average could top height by up to 200 m while the Δ*x* = 12 km simulations underestimate it by up to 400 m. Furthermore, the ensemble spread increases significantly in the hydrostatic runs with some members having very low cloud top heights. Similar characteristics are seen for maximum cloud top heights that focus on overshooting tops that are associated with the most intense updrafts (figure 6*i*). These results are consistent with the overestimation of vertical mass fluxes and updraft velocities in kilometre-scale simulations (see §3a(ii)).

Average cloud water mixing ratios agree well across grid spacings with the exception of higher mixing ratios close to the surface in the Δ*x* ≤ 2 km runs (electronic supplementary material, figure S2). Average rain water mixing ratios are significantly larger between 2 km and 7 km height for Δ*x* ≥ 500 m. The area with highest graupel mixing ratio around 6 km height is well simulated in simulations with Δ*x* ≤ 4 km and is significantly lower in the Δ*x* ≤ 12 km runs. Simulations with 500 m ≤ Δx ≤ 2 km overestimate graupel close to the surface. Snow mixing ratios in the anvil cloud (10–15 km height) are similar in simulations with Δ*x* ≤ 4 km but significantly larger in the Δ*x* = 12 km simulation. Ice mixing ratios are small in the Thompson microphysics scheme [37] but are systematically overestimated in all simulations compared to the Δ*x* = 250 m runs (electronic supplementary material, figure S2).

### (b) Grid spacing dependence of climate change signals

The second question that we asked in the introduction is how model Δ*x* affects the climate change signals of MCS processes, which is addressed in this section. Therefore, we regrid all simulations to the common grid of the Δ*x* = 12 km simulations and calculate ensemble mean climate change between the 10 member current and 10 member future idealized MCS ensembles. Statistics are calculated for each MCS case first and then averaged over the current and future ensemble using the ensemble spread to calculate statistical significance in future changes.

#### (i) Extreme precipitation climate change signals

**Figure 7.**
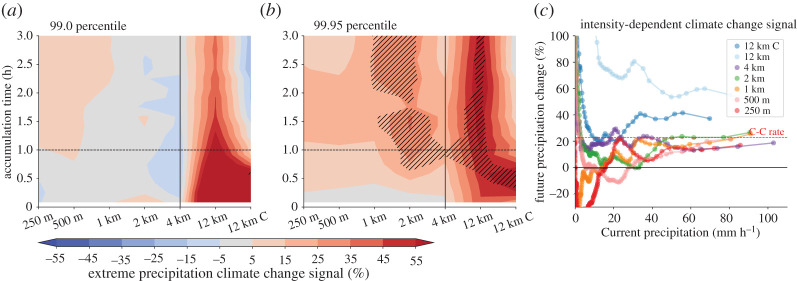
Extreme precipitation climate change signal dependence on horizontal grid spacing and accumulation period for the ensemble mean of 99 (*a*) and 99.95 (*b*) percentile values (including zero precipitation). Hatched areas show significant changes in the ensemble mean according to the Mann–Whitney test (*α* = 0.1). (*c*) Average relative climate change signal dependent on hourly precipitation intensity based on the ensemble mean hourly MCS precipitation during the hour with peak precipitation (including zero precipitation). (Online version in colour.)

Figure 7*a* shows the 99 percentile (P99; moderately intense precipitation of approx. 10 mm^−1^) change of precipitation for accumulation periods ranging from 5 min to 3 h, which represents moderate precipitation intensities. Most obvious is the large increase of P99 precipitation intensities in Δ*x* = 12 km runs in future climates especially for short accumulation periods. By contrast, simulations with Δ*x* ≤ 4 km do not show any noticeable change.

Extreme rainfall rates (99.95 percentile; P99.95) are projected to increase in all simulations and across all accumulation periods (figure 7*b*). Again, much larger increases are simulated in the Δ*x* = 12 km runs compared to higher resolution models. Not using a deep convection scheme at Δ*x* = 12 km results in very strong increases across all accumulation periods. The Δ*x* = 4 km runs produce very similar climate change signals compared to the Δ*x* = 250 m simulations while the Δ*x* = 2 km and Δ*x* = 1 km simulations project systematically approximately 10% higher extreme intensities for accumulations longer than 1 h. This indicates that there are compensating errors in the Δ*x* = 4 km simulations that enhance the agreement of their climate change projections compared to large eddy simulations and that there is no simple convergence of climate change signals with decreasing grid spacing.

Precipitation intensity-dependent climate change signals of hourly accumulations also show good agreement between kilometre-scale and sub-kilometre-scale simulations for extreme intensities (figure 7*c*). Hourly precipitation intensification in these simulations is consistent with saturation vapour increases, which is approximately 6.5% per degree warming according to the Clausius–Clapeyron (C–C) relationship [50]. Simulations with Δ*x* = 12 km, in contrast, result in much higher extreme intensification that can exceed twice the C–C relationship particularly in the simulations without deep convection scheme. Changes in weak and moderate hourly precipitation intensities are more variable than extremes.

Repeating the above analysis on the native model grid shows very similar extreme precipitation climate change signals (electronic supplementary material, figure S3). This confirms that extreme precipitation increases at approximately C–C rates in simulations with grid spacings Δ*x* ≤ 4 km even when localized extremes are considered.

#### (ii) Vertical mass flux climate change signals

**Figure 8.**
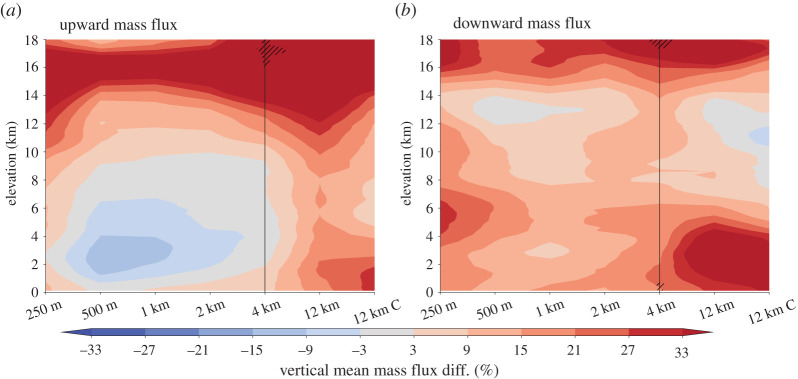
Ensemble mean MCS average up- (*a*) and downdraft (*b*) mass flux climate change signal dependence on horizontal grid spacing and height above surface. Hatched areas show significant changes in the ensemble mean according to the Mann–Whitney test (*α* = 0.1). (Online version in colour.)

Average upward mass flux increases by approximately 5% below 7 km height and by more than 33% above 12 km in the Δ*x* = 250 m simulations (figure 8*a*). This is consistent with the increase in CAPE at high altitudes in the inflow soundings (figure 2*c*) and generally deeper convection in the future climate (see next section). However, these changes are not significant due to the large variability in average mass flux in the current and future MCS ensemble. These changes are roughly captured in the Δ*x* ≤ 4 km simulations, whereas the Δ*x* = 12 km runs simulate a large increase in upward mass flux close to the surface.

Also average downward mass flux is increasing in the future Δ*x* = 250 m MCSs with maxima at approximately 5 km and above 16 km height. Simulations using Δ*x* between 500 m and 4 km capture this general pattern while the Δ*x* = 12 km simulations show a large increase in downward mass flux close to the surface instead of the mid troposphere (figure 8*b*).

#### (iii) Cold pool and cloud top climate change signals

**Figure 9.**
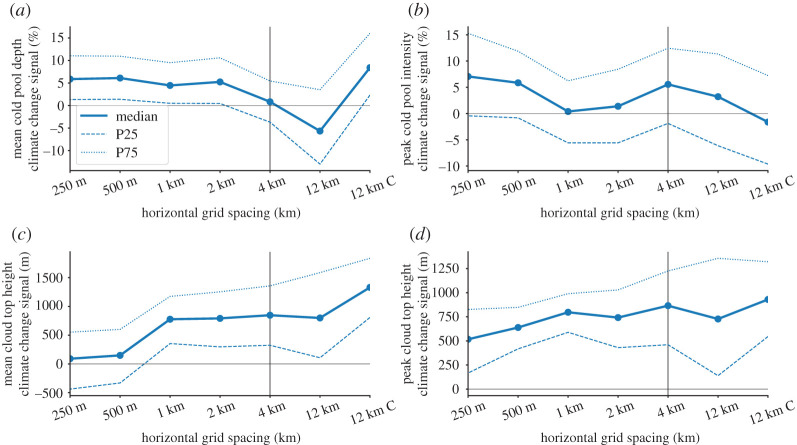
Ensemble climate change signals of mean cold pool depth (*a*), peak cold pool intensity (*b*), mean cloud top height (*c*) and maximum cloud top height (*b*) depended on horizontal grid spacing. The thick lines show the median and the thin dashed/dotted lines the 25/75 percentile spread of a 1000 member bootstrap sample with replacement. (Online version in colour.)

Cold pools systematically intensify under climate change and deepen approximately by 5% on average in the Δ*x* = 250 m simulations (figure 9*a*). Cold pool depth changes are similar in kilometre-scale simulations but changes are negative in Δ*x* = 12 km runs that do not use a deep convection scheme. Peak cold pool intensities also intensify by about 8% in the sub-kilometre scale simulations but kilometre-scale simulations show no change—except for the Δ*x* = 4 km runs that show an intensification. The Δ*x* = 12 km simulations show no systematic changes.

Average cloud top heights do slightly increase under future conditions in sub-kilometre-scale simulations (figure 9*c*). However, kilometre-scale and the Δ*x* = 12 km simulations without deep convection scheme how a robust deepening of the cloud top by approximately 700 m. Even stronger increases of approximately 1300 m are found in the Δ*x* = 12 km that include the Kain–Fritsch deep convection scheme. A similar but less pronounced sensitivity is present for increases in the peak cloud top height (i.e. overshooting tops; figure 9*c*). Those increase approximately by 550 m in the sub-kilometre and approximately 800 m in the coarser resolution simulations.

#### (iv) Microphysics climate change signals

**Figure 10.**
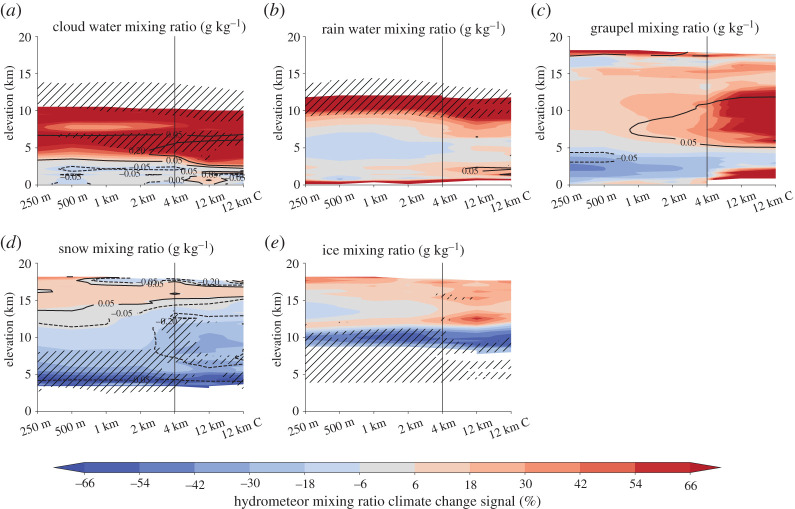
Ensemble mean hydrometeor mixing ratio climate change signals. Shown are results for in-cloud (particle mixing ration greater than 0.1 g kg^−1^) mixing ratios for horizontal cloud fields larger than 7.200 km^2^ for cloud water (*a*), rain (*b*), graupel (*c*), snow (*d*) and ice (*e*) for hour 1 to 7 after simulation start. Hatching shows significant differences (*α* = 0.05) and contour lines show absolute differences (in g kg^−1^). (Online version in colour.)

Hydrometeor mixing ratios change significantly under warming due to changes in thermodynamics and dynamics of future MCSs (figure 10). There is consensus across grid spacings that cloud water mixing ratios will not change in the lowest 3 km but will increase above up to approximately 10 km height (figure 10*a*). Increases in the mid troposphere are more systematic and intense in kilometre-scale models. A similar upward shift can be seen in rain water mixing ratio, which significantly increases approximately above 9 km and close to the surface (figure 10*b*). This upward shift is mainly due to an increase in the freezing level height and a larger saturation mixing ratio in future climates allowing higher concentrations of liquid particles (partly super-cool droplets) at higher altitude.

Large, but non-significant, grid spacing dependencies are shown for graupel mixing ratio showing much larger increases in low and mid levels in the Δ*x* = 12 km simulations than in the higher resolution simulations (figure 10*c*). Particularly the sub-kilometre simulations feature a loss of graupel at lower levels, which is likely driven by enhanced melting [54]. A similar but more significant melting loss at mid-levels can be seen for snow mixing ratio across all grid spacings up to Δ*x* = 4 km (figure 10*d*). Again, the Δ*x* = 12 km runs show different characteristics with larger losses at higher levels and smaller increases in the anvil cloud. Ice mixing ratio show a clear transition from significant decreases approximately below 11 km to increases above with decreases being fairly homogeneous across model resolutions while increases are larger at coarser grid spacings (figure 10*e*).

## 4. Summary and conclusion

We perform 10 member ensemble simulations of idealized MCSs under current and end-of-the-century climate conditions at six horizontal grid spacings (Δ*x*) ranging from hydrostatic-sales (Δ*x* = 12 km) to large eddy simulation scales (Δ*x* = 250 m). Several MCS bulk components such as MCS precipitation characteristics, cold pools, drafts and anvil clouds are compared across model resolutions on a common 12 km grid. The goal is to investigate systematic differences and convergence behaviours in the current climate and the effect of model grid spacing on climate change signals. We decided to focus on mean and extreme aspects of MCSs rather than spatial structures since the latter depends strongly on the initialization of the MCS.

**Figure 11.**
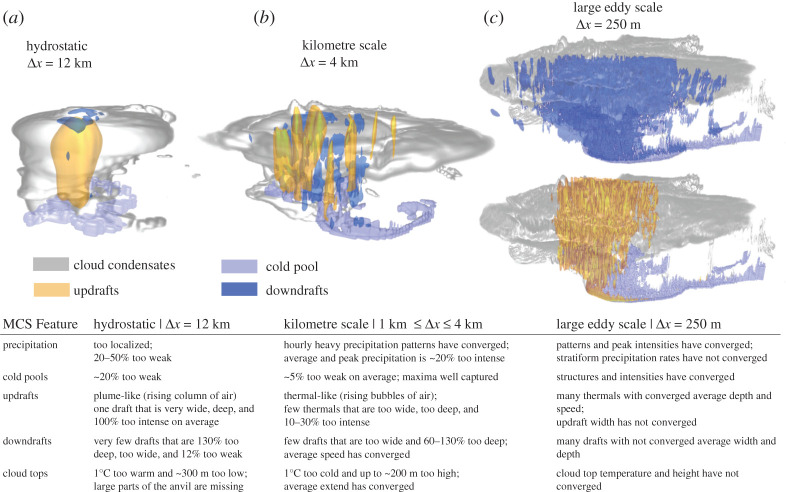
MCS features as simulated with hydrostatic (*a*, Δ*x* = 12 km), kilometre scale (*b*, Δ*x* = 4 km) and large eddy scale (*c*, Δ*x* = 250 m) horizontal grid spacing based on a representative example MCS. Shown are the cloud condensates (grey shading), cold pools (violet; −0.005 m s^−2^), updrafts (orange; more than 3 m s^−1^) and downdrafts (blue; less than −1.5 m s^−1^). The vertical axis is stretched by a factor of 10. The table summarizes the main differences between features simulated by hydrostatic and kilometre-scale runs compared to those in thelarge eddy simulations. (Online version in colour.)

Figure 11 shows the main differences of MCS features as simulated with hydrostatic, non-hydrostatic and large eddy grid spacings.

The following conclusions summarize this paper:

— There is a step improvement in simulating MCSs when increasing Δ*x* from hydrostatic (12 km) to non-hydrostatic (less than or equal to 4 km) grid spacings. The Δ*x* = 4 km simulations can reproduce most of the salient MCS features, such as track, maximum precipitation, cold pool intensity and cloud top temperatures from the Δ*x* = 250 m runs within ±20% at 0.02% of their computational costs and 0.4% of their output volume. These results are in agreement with existing MCS literature [12,23,55].
— Minor differences occur between simulations with Δ*x* = 4 km and 1 km when compared on a common 12 km grid. Higher resolution simulations are able to simulate small-scale processes, such as up- and downdraft width and depth, more realistically [46] but this added value has only minor effects on MCS bulk processes.
— A clear deficit of kilometre-scale models is an overestimation of draft velocities and convective mass flux of up to 30% compared to the Δ*x* = 250 m simulations. As a consequence, kilometre-scale models simulate approximately 20% higher peak rainfall rates and higher and cooler cloud tops. This is likely related to an underestimation of entrainment in kilometre-scale models due to under-resolved turbulent processes [15,19] and a misrepresentation of non-hydrostatic effects [23].
— Recent studies show some benefits in not using deep convection schemes for models with Δ*x* > 10 km [56]. We confirm these results and show clear advantages in the Δ*x* = 12 km simulations without deep convection schemes compared to the ones with deep convection schemes under current climate conditions (although large differences to the Δ*x* ≤ 4 km simulations still exist). The benefits of not using a deep convection scheme will likely decrease with increasing Δ*x* and key deficiencies (i.e. potential build up of large buoyancy) will start to dominate. Care should be taken since the absence of a deep convection scheme at Δ*x* = 12 km results in much larger increases in vertical mass fluxes and extreme precipitation under future climate conditions.
— Most important for climate modelling is that climate change signals in kilometre-scale simulations agree much better with Δ*x* = 250 m simulations than those from Δ*x* = 12 km runs. However, important differences such as a significant overestimation of extreme rainfall rates in 1 km and 2 km simulations remain, whereas the better agreement in 4 km simulations is likely due to compensating errors. The nature of these compensating errors and the development of scale aware parametrization schemes to mitigate systematic deficiencies in kilometre-scale models should be the focus of future research.

The future MCS environmental conditions are based on simulations that use the pseudo global warming approach assuming that synoptic-scale weather patterns do not change systematically under global warming. We believe that this assumption does not change the main conclusions of this study since it is plausible that future intense MCSs will develop in similar environmental conditions as in the current climate (i.e. moderate wind shear and CAPE, high column average relative humidity, moderate to low CIN). The main differences are an increase in precipitable water, CAPE and atmospheric stratification, which are captured in the PGW approach [57]. The PGW approach might provide less reliable results for changes in MCS frequencies and shifts in their seasonality, which are not the focus in this study.

A caveat of this study is the small sample size (10-current and future MCSs) complicates a robust assessment of climate change effects. Furthermore, results using more realistic MCS simulations might differ from idealized results since MCSs in the USA frequently develop along atmospheric boundaries (e.g. fronts, drylines) that affect the organization and evolution of MCSs [58]. Additionally, land-surface heterogeneities can affect the initiation and development of convection [59]. Both of these effects might reduce the sensitivity of MCSs to horizontal grid spacing since these features or heterogeneities provide external forcing that can be captured in kilometre-scale models. We also neglected the impact of radiation and planetary boundary layer effects in our idealized simulations. These effects are likely better represented at LES scales and might increase the sensitivity to horizontal grid spacing. Future research will address these open questions by simulating observed MCSs in the US Southern Great Plains and the Amazon basin. Better understanding the impacts of environmental conditions and model resolution on simulating MCSs is important since the frontier of global-atmospheric modelling has reached kilometre scales [60,61].

## Data accessibility

All scientific code that was used in this study can be accessed through GitHub [25]. The WRF modelling system is open source and is available for download from https://github.com/wrf-model/WRF. The sounding data that is used to initialize the idealized MCS simulations are available from https://issues.pangaea.de/browse/PDI-23519 [62]. The complete ensemble simulation dataset has a data volume of 39 TB and can be accessed by request from the corresponding author.
